# Attenuation of Experimental Colitis in Glutathione Peroxidase 1 and Catalase Double Knockout Mice through Enhancing Regulatory T Cell Function

**DOI:** 10.1371/journal.pone.0095332

**Published:** 2014-04-17

**Authors:** Hyung-Ran Kim, Anbok Lee, Eun-Jeong Choi, Jeong-Hae Kie, Woosung Lim, Hyeon Kook Lee, Byung-In Moon, Ju-Young Seoh

**Affiliations:** 1 Department of Microbiology, Ewha Womans University Graduate School of Medicine, Seoul, Korea; 2 Department of Pathology, National Health Insurance Cooperation Ilsan Hospital, Koyang, Korea; 3 Department of Surgery, Ewha Womans University Graduate School of Medicine, Seoul, Korea; Université Paris Descartes, France

## Abstract

Reactive oxygen species (ROS) have been implicated in the progression of inflammatory diseases including inflammatory bowel diseases (IBD). Meanwhile, several studies suggested the protective role of ROS in immune-mediated inflammatory diseases, and it was recently reported that dextran sodium sulfate (DSS)-induced colitis was attenuated in mice with an elevated level of ROS due to deficiency of peroxiredoxin II. Regulatory T cells (Tregs) are critical in the prevention of IBD and Treg function was reported to be closely associated with ROS level, but it has been investigated only in lowered levels of ROS so far. In the present study, in order to clarify the relationship between ROS level and Treg function, and their role in the pathogenesis of IBD, we investigated mice with an elevated level of ROS due to deficiency of both glutathione peroxidase (GPx)-1 and catalase (Cat) for the susceptibility of DSS-induced colitis in association with Treg function. The results showed that DSS-induced colitis was attenuated and Tregs were hyperfunctional in GPx1^−/−^ × Cat^−/−^ mice. In vivo administration of N-acetylcysteine (NAC) aggravated DSS-induced colitis and decreased Treg function to the level comparable to WT mice. Attenuated Th_17_ cell differentiation from naïve CD4^+^ cells as well as impaired production of IL-6 and IL-17A by splenocytes upon stimulation suggested anti-inflammatory tendency of GPx1^−/−^ × Cat^−/−^ mice. Suppression of Stat3 activation in association with enhancement of indoleamine 2,3-dioxygenase and FoxP3 expression might be involved in the immunosuppressive mechanism of GPx1^−/−^ × Cat^−/−^ mice. Taken together, it is implied that ROS level is critical in the regulation of Treg function, and IBD may be attenuated in appropriately elevated levels of ROS.

## Introduction

Reactive oxygen species (ROS) are highly reactive and interact with many bio-molecules. At high concentrations, they are likely to destroy biological structures, promoting cellular damage and tissue destruction. Traditionally, ROS have been implicated in ageing and the progression of inflammatory and autoimmune diseases, including inflammatory bowel diseases (IBD) [1], [2], [3]. Meanwhile, many recent observations are opposing the traditional concept on ROS, suggesting the protective role of ROS in immune-mediated inflammatory diseases [4].

Mice with lower level of ROS than WT mice due to defects in ROS-producing enzyme system, such as Ncf1^−/−^ or Nox2^−/−^, are more susceptible to autoimmune diseases, such as arthritis and encephalomyelitis [5], [6], [7]. Humans with lower levels ROS than normal persons, such as chronic granulomatous disease (CGD) patients and carriers, are also more susceptible to autoimmune diseases [8], [9]. By contrast, mice with higher level ROS than WT mice due to the defect in a ROS metabolizing enzyme, glutathione peroxidase-1 (GPx-1), are resistant to immune-mediated inflammatory diseases, such as allergen-induced airway inflammation and high fat diet-induced atherosclerosis [10], [11]. In particular, mice with higher level of ROS due to defect of a non-enzymatic cellular anti-oxidant, peroxiredoxin (Prx) II, are resistant to dextran sodium sulfate (DSS)-induced colitis [12].

These clinical or experimental observations implicated the immunoregulatory role of ROS, and adoptive-transfer of CD4^+^ cells from rats with lower ROS level induced arthritis in rats with normal ROS level, demonstrating the key role of CD4^+^ cells in the hyperinflammatory response in lowered levels of ROS [13]. On the other hand, oxidative stress induces T cell hyporesponsiveness in several human pathologies (*e.g.* cancer, rheumatoid arthritis, AIDS and leprosy) [14], [15]. Accordingly, ROS level is supposed to be closely associated with T cell responsiveness. In particular, regulatory T cell (Treg) function seems to be closely linked to ROS level. Tregs isolated from mice with lower level of ROS, such as Ncf1^−/−^ mice, were hypofunctional than WT Tregs [16]. Tregs were also hypofunctional in vitro at lowered levels of ROS by adding antioxidants or NADPH oxidase inhibitors. Differentiation of inducible Treg (iTreg) seems also linked to the level of ROS. Induction of FoxP3^+^ iTreg was attenuated, whereas that of Th_17_ cells was enhanced in lowered levels of ROS due to Nox2 deficiency [6], [7] or addition of apocynin [17]. By contrast, induction of FoxP3^+^ Treg was enhanced in elevated levels of ROS due to PrxII deficiency [12]. Meanwhile, the suppressive function of Tregs has been investigated only in lowered levels of ROS so far, and the suppressive function of GPx1^−/−^ or PrxII^−/−^ Tregs has not yet been reported.

Thus, in the present study, we investigated the suppressive function of Tregs isolated from mice with elevated levels of ROS due to defects in GPx1 and catalase (Cat) [18]. The results showed that GPx1^−/−^ × Cat^−/−^ Tregs were hyperfunctional and GPx1^−/−^ × Cat^−/−^ mice were resistant to DSS-induced colitis. Meanwhile, administration of n-acetylcysteine (NAC) reduced Treg function and made GPx1^−/−^ × Cat^−/−^ mice susceptible to DSS-induced colitis.

## Materials and Methods

### Mice

C57BL/6 wild-type (WT) and GPx1^−/−^ × Cat^−/−^ mice with a C57BL/6 genetic background were housed and maintained in the animal facility at Ewha Womans University [18]. This study was performed according to Korean Food and Drug Administration guidelines and was specifically approved by the Institutional Animal Care and Use Committee of Ewha Womans University Graduate School of Medicine (Permit Number: 10-0133).

### ROS Measurement

Ten million splenocytes prepared by mincing from WT or GPx1^−/−^ × Cat^−/−^ mice were incubated with 5 µM dichloro-fluoroscein diacetate (DC-FDA, Sigma, St. Louis, MO) for 30 min at 4°C in the dark. For the surface stain, anti-CD4-PerCP and anti-CD25-PE or anti-CD11c-PE purchased from BD Biosciences (San Diego, CA) were incubated together. The cells were washed twice with cold PBS and were suspended in DMEM. Then, the cells were analyzed with a FACSCalibur for fixed time. After 30 second from start of data reading, the cells were stimulated with 100 nM phorbol 12-myristate 13-acetate (PMA, Calbiochem, Darmstadt, Germany).

### Induction of Acute Experimental Colitis

Males at 8–10 weeks of age were administered with 3% dextran sodium sulfate (DSS) purchased from MP Biomedicals (Strasbourg, France) in drinking water for 4 days. During the 4 days, the mice were restricted from water supply for 12 hours and then fed with 3% DSS water for 12 hours in a day. Each mouse drank about 20 mL of 3% DSS water during the 4 days. Some mice were administered intra-gastrically with 400 µL of 40 mM N-acetylcysteine (NAC, Sigma) in water, as mice do not like the flavor of NAC, every day from 3 days before the treatment with DSS to the end of the experiment, except for the 4 days during which DSS was administered. Body weight was measured every day and the mice were sacrificed on the 7^th^ day after treatment with DSS. For histological examination, the colons were removed, rolled around a cotton swab and were fixed in 10% formaldehyde to be embedded in paraffin. The paraffin blocks were longitudinally sectioned serially with the thickness of 5 µm and were stained with hematoxylin and eosin (H&E) to allow histological examination of whole colons.

### Immunohistochemistry

Sections were deparaffinated in xylene, dehydrated in ethanol and washed in PBS followed by successive permeabilization steps (with Triton 0.2% in PBS). Endogenous peroxidase was blocked with hydrogen peroxide (5% in PBS) for 30 min and the sections were subjected to heat-induced antigen retrieval step before incubation with a universal blocking solution (Dako, Glostrup, Denmark) for 30 min. Then, the sections were incubated with anti-phosphotyrosine (pY)-Stat3 (Tyr705) (clone D3A7, Cell Signaling Technology, Danvers, MA), anti-indoleamine 2,3,-dioxygenase (IDO, rabbit polyclonal antibody, Abcam, Cambridge, UK) or anti-FoxP3 (rabbit polyclonal antibody, Abcam). Then, the sections were incubated with labelled streptavidin biotin reagents for rabbit, mouse and goat antibodies (Dako, Glostrup, Denmark) and developed using 3,3′-diaminobenzidine as chromogen substrate. For the counting of FoxP3^+^ cells, 5 high-power fields selected at random in the lesions in each slide were examined by three different pathologists.

### Preparation of Cells

Spleens were removed from sacrificed mice and single cell suspension was prepared by squeezing on a cell strainer (70 µm, BD Biosciences, San Jose, CA). After erythrocytes were lysed with RBC lysis buffer (eBioscience, San Diego, CA), CD4^+^CD25^+^ fraction was separated using a regulatory T cell isolation kit purchased from Miltenyi Biotech (Auburn, CA). CD4^+^CD25^−^ cells were also isolated and used for effector T cells (Teffs). For the purity check, the cells were stained for surface CD4 and CD25, followed by intranuclear staining for FoxP3. After Fc receptors were blocked using anti-mouse CD16/CD32 (2.4G2, BD Biosciences) for 15 min at 4°C, cells were incubated with anti-CD4-FITC (H129.19) and anti-CD25-PE (PC61) purchased from BD Biosciences for 30 min at 4°C. After washed, the cells were fixed and permeabilized using a mouse regulatory T cell staining kit (eBiosciences, San Diego, CA) and were stained with anti-Foxp3-PerCP-Cyanine5.5 (FJK-16 s). The stained cells were analyzed using a FACSCalibur (BD Biosciences). Teffs were labeled with carboxyfluorescein diacetate succinimidyl ester (CFSE, Invitrogen, Carlsbad, CA) as described elsewhere, to trace the proliferative response [19]. For the purification of dendritic cells (DCs), CD11c^+^ cells were isolated using a spleen dissociation medium (StemCell technologies, Vancouver, BC), density gradient centrifugation over 15.5% Accudenz (Accurate Chemical & Scientific, Westbury, NY), and immunomagnetic selection using anti-CD11c-PE (BD Biosciences) and microbead anti-PE (Miltenyi Biotec). The purity of CD11c^+^ cells was consistently over 95%. Sometimes, cells were prepared 24 hours after the intraperitoneal injection of NAC (10 mg in PBS) or PBS.

### *In vitro* Suppressive Assay

For the functional assessment of the suppressive activity of Tregs, DCs, Teffs and Tregs were co-cultured in the ratio of 0.2∶1∶1. Meanwhile, a small percentage of CD4^+^CD25^+^ T cells are not Tregs and do not express FOXP3, and the isolated Treg samples were not pure based on FOXP3 expression. In the present study, 86.6∼91.4% of the purified Treg fractions were CD4^+^FoxP3^+^ (Fig. S1). To ensure optimal reproducibility of the suppression assays, the degree of purity of the Treg samples was taken into account when the cells were plated so that the final ratio of CD4^+^FOXP3^+^ Tregs to CD4^+^FOXP3^−^ Teff was as close as possible to 1∶1 [16]. For the proliferative responsiveness, 10^4^ CFSE-labeled CD4^+^FOXP3^−^ Teffs and 2×10^3^ DCs were cultured in DMEM supplemented with 10% FCS (Hyclone, Logan, UT) in round-bottomed 96-well plates by stimulating with various concentrations of soluble anti-CD3e (e-Biosciences). On the 3^rd^ day of culture, the cells were harvested for staining with anti-CD4-PerCP. Whole cells were acquired by using a FACSCalibur and were analyzed by using Winlist software (Verity, Topsham, ME). Precursor frequency (Pf) was estimated for the cells exclusively gated for CD4^+^ live cells according to the scattering characteristics, using the proliferation wizard of Modifit software (Verity), as described elsewhere [19], [20]. In order to assess the suppressive function of Tregs, CD4^+^FOXP3^+^10^4^ Tregs were added to the culture, and the Pf values in the absence and presence of Tregs were compared to give rise to suppression (%). In order to compare the suppressive function of WT Tregs and GPx1^−/−^ × Cat^−/−^ Tregs, feeder function of WT DCs and GPx1^−/−^ × Cat^−/−^ DCs, and the proliferative activity of WT Teffs and GPx1^−/−^ × Cat^−/−^ Teffs, 3 kinds of cells (DCs, Teffs and Tregs) in the culture from the WT and GPx1^−/−^ × Cat^−/−^ mice were cross-combined to give rise to 8 kinds of combinations, as shown in Fig. S2. When necessary, catalase (100 U/mL, Sigma) or NAC (40 nM) were added to the cultures.

### In vitro Th_17_/Treg Cell Differentiation

Naïve CD4^+^ T cells were isolated from the spleen of WT or GPx1^−/−^ × Cat^−/−^ mice using a naive CD4^+^ T cell isolation kit (R&D Systems, Minneapolis, MN). For the induction of Th_17_ cell differentiation, 1×10^5^ naïve CD4^+^ cells were stimulated with soluble anti-CD3e (1 µg/mL) and soluble anti-CD28 (1 µg/mL, e-Biosciences) in the presence of 2×10^4^ CD11c^+^ DCs for 24 hours, and were cultured further for 2.5 days in the presence of TGF-β1 (5 ng/mL) and IL-6 (20 ng/mL) purchased from R&D Systems. For intracellular cytokine staining, cells were re-stimulated for 4 hr with PMA (25 ng/mL) and ionomycin (250 ng/mL, Sigma) in the presence of a protein trapsport inhibitor containing monensin (BD Biosciences). Then, the cells were harvested and stained for intracellular IL-17A and IFN-γ using a commercial kit for fixation and permeabilization ((BD Biosciences), anti-mouse IL-17A-PE (eBioscience) and anti-mouse IFN-γ-FITC (eBioscience). For the induction of iTreg differentiation, 1×10^5^ naïve CD4^+^ cells were stimulated with plate-coated anti-CD3e (50 ng/well) and soluble anti-CD28 (1 µg/mL) in the presence of TGF-β1 (5 ng/ml) and human recombinant IL-2 (10 U/mL, BD Biosciences). After three days of culture, the cells were harvested for intranuclear staining for FoxP3, using a mouse regulatory T cell staining kit.

### Cytokine Production Assay

In order to evaluate the producing capability of inflammatory cytokines, IL-6 and IL-17A, 10^6^ splenocytes were stimulated with soluble anti-CD3e (1 µg/mL) and anti-CD28 (1 µg/mL). After 5 days of culture, cytokines secreted in the supernatants were analyzed by cytometric bead array (BD Biosciences). Briefly, the culture supernatants were mixed with anti-mouse IL-6, anti-mouse IL-17A capture beads and PE detection reagent for 2 hr at RT. The beads were washed with 1 mL of wash buffer and re-suspended in 300 µL of wash buffer, and were analyzed with appropriate acquisition template supplied by BD Biosciences.

### Statistical Analysis

Data are expressed as mean±SE of more than six separate experiments. Comparison of data was done by using independent Student’s t test or ANOVA. *P* values less than 0.05 were considered statistically significant.

## Results

### Intracellular ROS Level was Higher in GPx1^−/−^ × Cat^−/−^ than WT Lymphocytes

**Figure 1:**
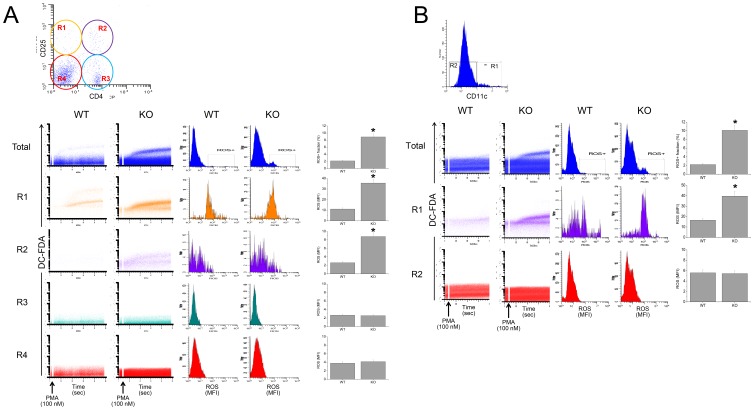
Intracellular ROS level was higher in GPx1^−/−^× Cat^−/−^ than WT lymphocytes. Splenocytes were stained with DC-FDA (5 µM) and simultaneously with anti-CD4-PerCP and anti-CD25-PE (A) or anti-CD11c-PE (B). Then the cells were washed and acquired using FACSCalibur. After stabilization for 30 sec, acquisition was briefly suspended and PMA (100 nM) was added to the tube, and the cells were acquired further for 482 sec. ROS level was expressed as mean fluorescence intensity (MFI) of DC-FDA fluorescence. Data are mean ± SE (n = 12). *, P<0.05.

Flow cytometric analysis of the splenocytes stained with DC-FDA after stimulation with PMA showed that the intracellular ROS level in the splenocytes from GPx1^−/−^ × Cat^−/−^ mice was higher than that from WT mice (Fig. 1A). Among the T cells used in the suppression assay, intracellular ROS level was higher in CD4^+^CD25^+^ Tregs than in CD4^+^CD25^−^ Teffs. Intracellular ROS level was higher in GPx1^−/−^ × Cat^−/−^ CD11c^+^ DCs than that in WT DCs (Fig. 1B).

### DSS-induced Colitis was Attenuated in GPx1^−/−^ × Cat^−/−^ Mice

**Figure 2:**
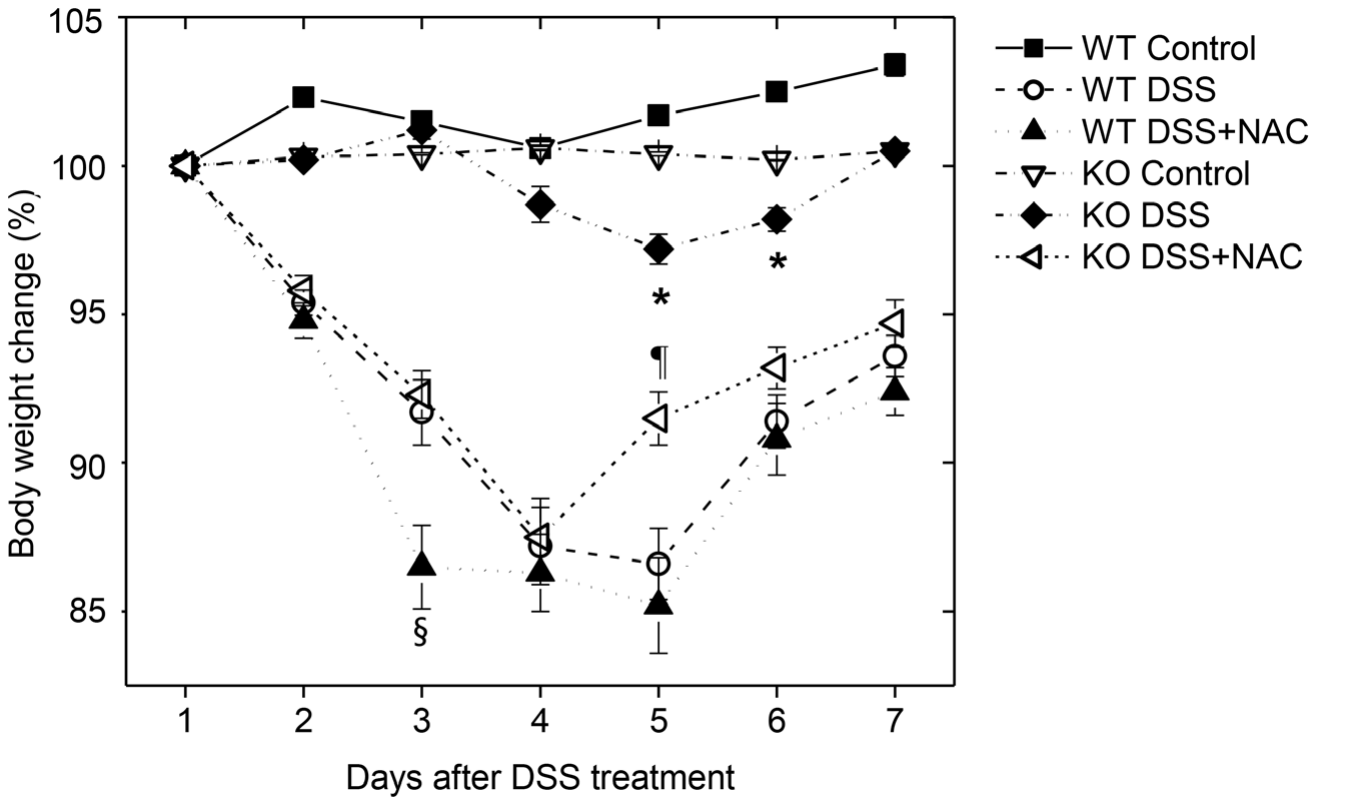
Body weight change during the course of DSS-induced colitis. Body weight of the mice was measured every day, after 4 days of oral administration of 3% DSS in drinking water. Some mice were administered intra-gastrically with 400 µL of 40 mM NAC in water, as mice do not like the flavor of NAC, every day from 3 days before the treatment with DSS to the end of the experiment, except for the 4 days during which DSS was administered. KO, GPx1^−/−^ × Cat^−/−^. Data are mean ± SE, n = 12 in each group. *, P<0.05, compared between KO mice control and KO mice treated with DSS. §, P<0.05, compared between WT mice treated with DSS+NAC and WT mice treated with DSS only. ¶, P<0.05, compared between KO mice treated with DSS and WT mice treated with DSS.

**Figure 3:**
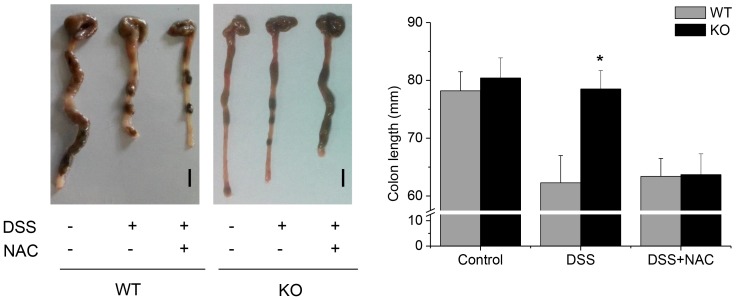
Colon length was not shortened in KO mice treated with DSS only, but was shortened with DSS and NAC. On the 7^th^ day after treatment with DSS, colon length was measured. KO, GPx1^−/−^ × Cat^−/−^. Scale bar is 1 cm. Data are mean ± SE, n = 12 in each group. *, P<0.05.

**Figure 4:**
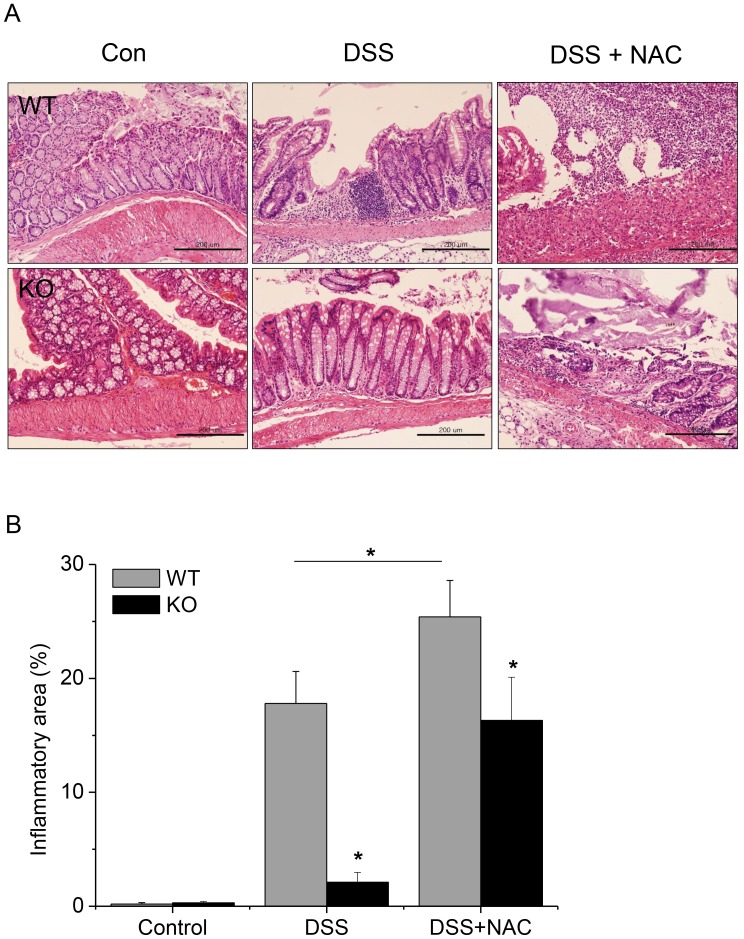
Inflammatory reaction was attenuated in the lesions of DSS-induced colitis in KO mice, but was aggravated by NAC. On the 7^th^ day after oral administration of NAC, the colons were removed, rolled around a cotton swab and were fixed to be embedded in paraffin. The paraffin blocks were longitudinally sectioned and were stained with H&E to allow histological examination of the whole colons (A). Scale bar is 200 µm. Inflammatory area was measured by histological examination of the entire colon by 3 separate pathologists (B). KO, GPx1^−/−^ × Cat^−/−^. Data are mean ± SE, n = 12 in each group. *, P<0.05.

**Figure 5:**
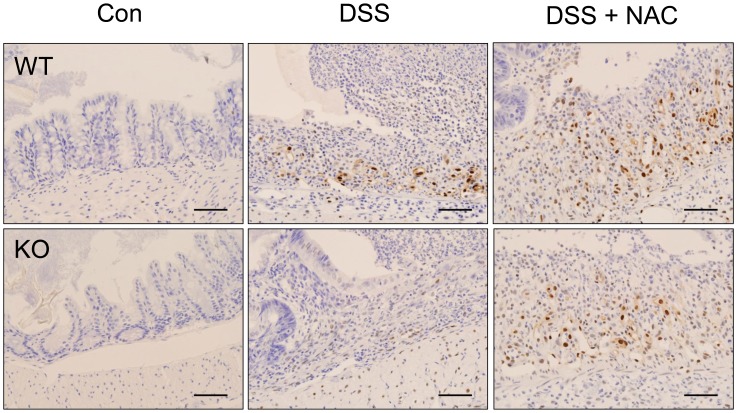
Stat3 was activated in the lesions of DSS-induced colitis in WT mice but not in KO mice. Immunohistochemical staining for phosphotyrosine (pY)-Stat3 (Tyr705) shows stat3 was activated in the lesions of DSS-induced colitis in WT mice. In the colons of KO mice treated with DSS, pY-Stat3 is observed but at very weak signal intensity in the infiltrating cells. Meanwhile, in the KO mice treated with both DSS and NAC, pY-Stat3 is observed as strongly as WT mice treated with DSS in the infiltrating cells. Scale bar is 50 µm.

After treatment with DSS, WT mice exhibited diarrhea, bloody stool and significant weight loss (Fig. 2), and colon length was significantly shortened (Fig. 3). Histological examination showed inflammatory changes in substantial area (17.8±4.2%, n = 12) of the entire colon, including inflammatory cell infiltration, severe ulceration, cryptic distortion and dysplastic changes (Fig. 4). Immunohistochemistry (IHC) showed enhanced expression of pY-Stat3 in the infiltrating cells as well as in the intestinal epithelial cells in the lesions of DSS-induced colitis (Fig. 5). By contrast, GPx1^−/−^ × Cat^−/−^ mice did not exhibit diarrhea, and weight loss was very slight only on the 5^th^ and 6^th^ day after treatment with DSS (Fig. 2). At the time of investigation on the 7^th^ day after treatment with DSS, colon length was not shortened (Fig. 3), and histological examination showed minimal inflammatory changes at restricted area (2.1±0.8%) (Fig. 4). Expression of pY-Stat3 was observed in the infiltrating cells but at very weak signal intensity (Fig. 5).

### Administration of NAC Aggravated DSS-induced Colitis

Administration of NAC before and after treatment with DSS into GPx1^−/−^ × Cat^−/−^ mice induced severe weight loss and colon shortening comparable to DSS-induced colitis in WT mice (Figs. 2 & 3). Meanwhile, they recovered earlier than WT mice treated with DSS (Fig. 2). Histological examination showed severe inflammatory changes and expression of pY-Stat3 comparable to DSS-induced colitis in WT mice (Fig. 4 & 5). Administration with both NAC and DSS induced earlier weight loss in WT mice than those treated with DSS only (Fig. 2). Histological examination showed more severe inflammatory changes in wider area in WT mice treated with both NAC and DSS (Fig. 4). Taken together, in vivo administration of NAC aggravated DSS-induced colitis both in WT as well as in GPx1^−/−^ × Cat^−/−^ mice.

### GPx1^−/−^ × Cat^−/−^ Tregs were Hyperfunctional

**Figure 6:**
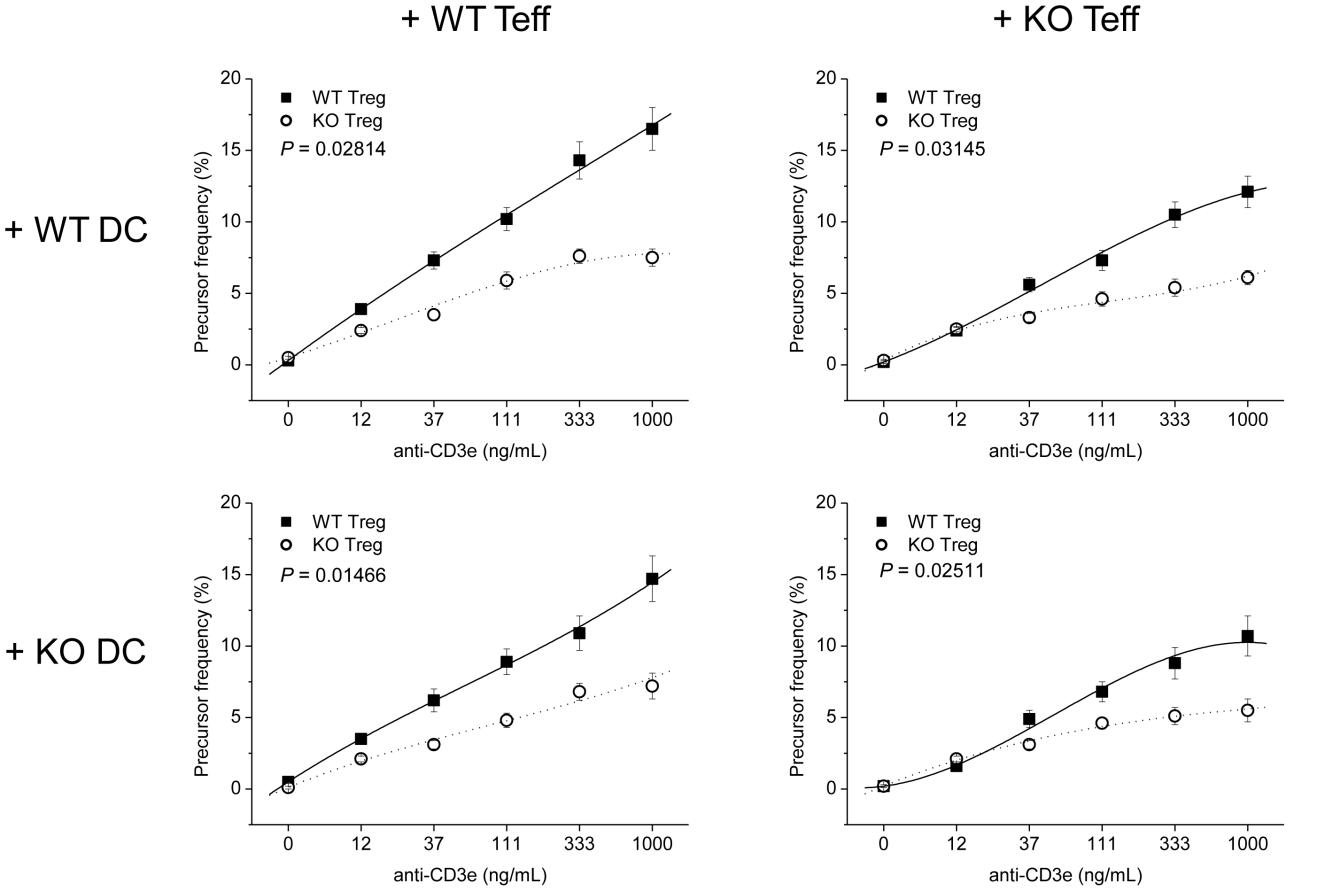
KO Tregs were hyperfunctional. CFSE-labeled Teffs were stimulated in the presence of DCs and Tregs from WT or KO mice. On the 3^rd^ day, the cells were harvested and stained for surface CD4. Live CD4^+^CFSE^+^ cells were gated for the analysis of the proliferative responsiveness of Teffs. The proliferative response of Teffs in the presence of KO Tregs was less active than in the presence of WT Tregs, suggesting KO Tregs were hyperfunctional in the suppression of Teffs than WT Tregs. KO, GPx1^−/−^ × Cat^−/−^. Data are mean ± SE of six separate experiments.

**Figure 7:**
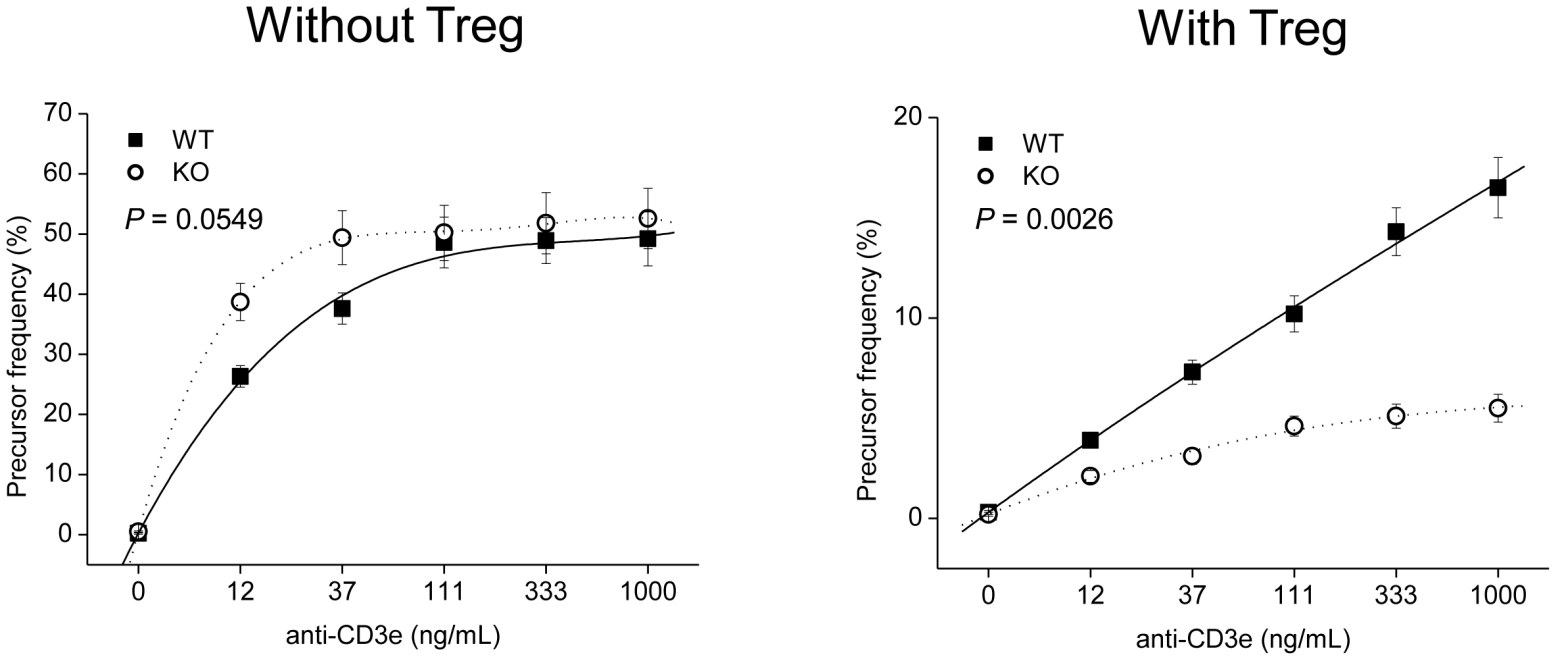
Simulated immune responsiveness of KO mice. Comparison of Teff proliferative responsiveness in the cultures of cognate cells from the same mice, i.e., between WT Teff+WT DC (+ WT Treg) and KO Teff+KO DC (+ KO Treg). In the absence of Tregs, KO Teffs were hyperproliferative, but in the presence of Tregs, were hypoproliferative than WT Teffs. KO, GPx1^−/−^ × Cat^−/−^. Data are mean ± SE of six separate experiments.

Teffs in the cocultures with KO Tregs was less proliferative than in cocultures with WT Tregs, suggesting KO Tregs were hyperfunctional in the suppression of Teff proliferation than WT Tregs (Fig. 6 & Fig. S2). Meanwhile, in the absence of Tregs, GPx1^−/−^ × Cat^−/−^ Teffs were slightly more proliferative than WT Teffs in the presence of WT CD11c^+^ DCs, not in the presence of GPx1^−/−^ × Cat^−/−^ DCs (Fig. S3 & Fig. S4). We also compared the proliferative activity of Teffs in the cocultures with cognate cells from the same mice, i.e., between WT Teff+WT DC (+ WT Treg) and KO Teff+KO DC (+ KO Treg) for the simulation of whole immune responsiveness of mice. The result showed KO Teffs were hyperproliferative in the absence of Tregs, but were hypoproliferative in the presence of Tregs, than WT Teffs (Fig. 7). Taken together, the attenuated DSS-induced colitis in GPx1^−/−^ × Cat^−/−^ mice might reflect the hypoactive immune responsiveness in the presence of Tregs, suggesting the critical role of Tregs in the regulation of immune responsiveness in vivo.

### Treg Function was Closely Associated with ROS Level

**Figure 8:**
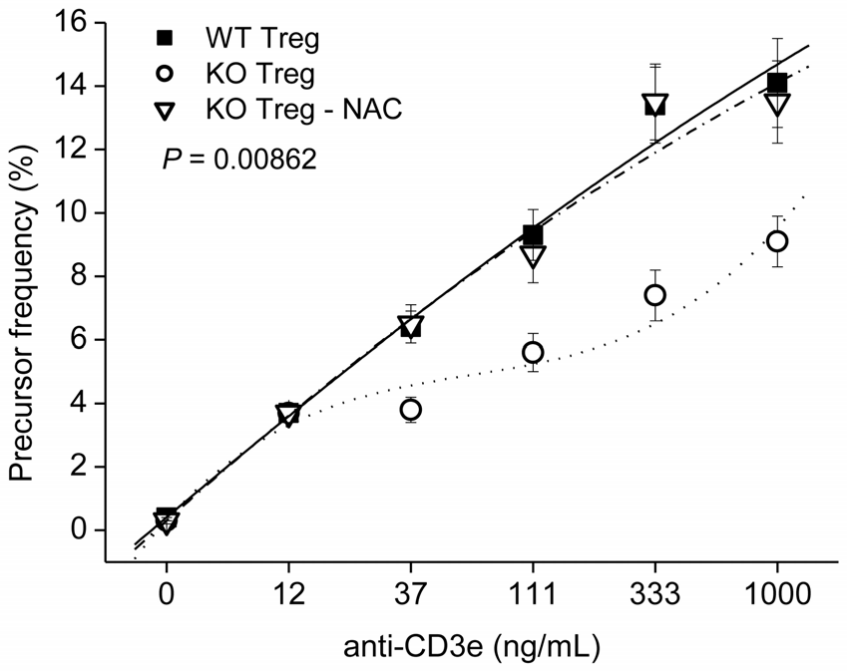
In vivo administration of NAC into KO mice reduced Treg function. Twenty four hours after the intraperitoneal injection of NAC (10 mg in PBS) or PBS (control), cells were prepared and analyzed for the suppressive function of Tregs in the same way as in Fig. 6. KO, GPx1^−/−^ × Cat^−/−^. Data are mean ± SE of six separate experiments. *P* value indicates significance of difference between KO Treg and KO Treg-NAC.

**Figure 9:**
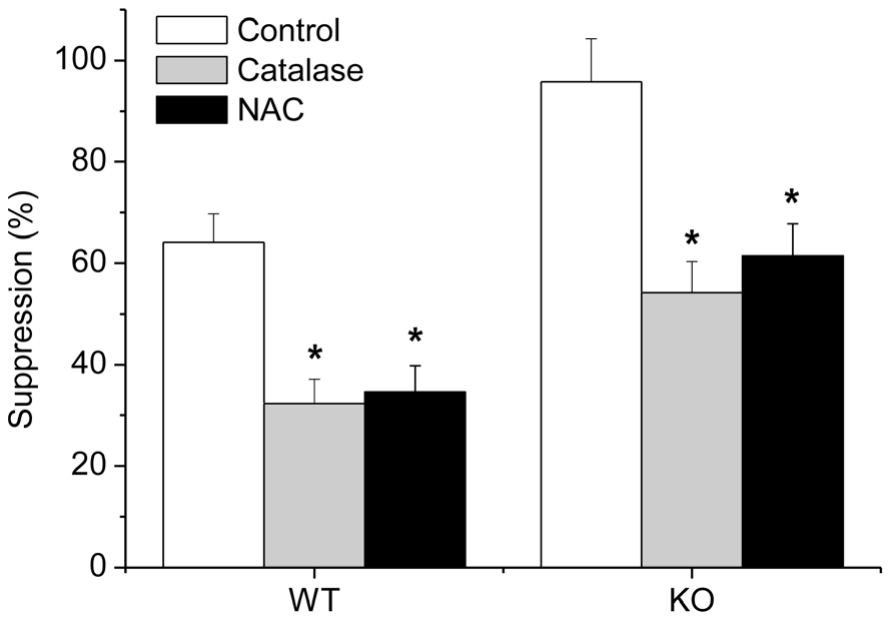
N-acetylcysteine (NAC) or catalase reduced Treg function in vitro. Comparison of Teff proliferation in the absence and presence of Tregs in response to soluble anti-CD3e (1 µg/mL) gave rise to suppression (%). Addition of NAC (40 nM) or catalase (100 U/mL) reduced the suppressive function of WT and KO Tregs in vitro. KO, GPx1^−/−^ × Cat^−/−^. Data are mean ± SE of six separate experiments.

The suppressive activity of Tregs isolated from GPx1^−/−^ × Cat^−/−^ mice one day after intraperitoneal (IP) injection of NAC was reduced to the level comparable to WT Tregs (Fig. 8). IP injection of NAC into WT mice also reduced the suppressive function of Tregs (data not shown). In addition, IP injection of vitamin C (500 mg/kg) also reduced the suppressive function of Tregs, suggesting the critical role of ROS level in Treg function (data not shown). Addition of NAC or catalase into the cultures also reduced the suppressive function of Tregs in vitro, providing another supportive evidence that ROS level is critical in the regulation of Treg function (Fig. 9).

### Anti-inflammatory Tendency of GPx1^−/−^ × Cat^−/−^ Mice

**Figure 10:**
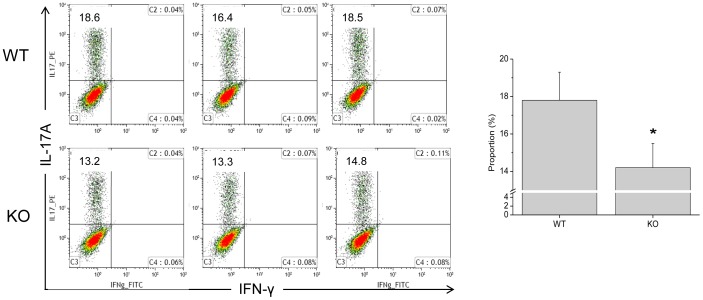
Th_17_ cell differentiation was mitigated in KO mice. Naïve CD4^+^ cells isolated from the spleens of WT or KO mice were induced to differentiate into Th_17_ cells by stimulating in the presence of TGF-β1 and IL-6. KO, GPx1^−/−^ × Cat^−/−^. Data are mean ± SE of six separate experiments.

**Figure 11:**
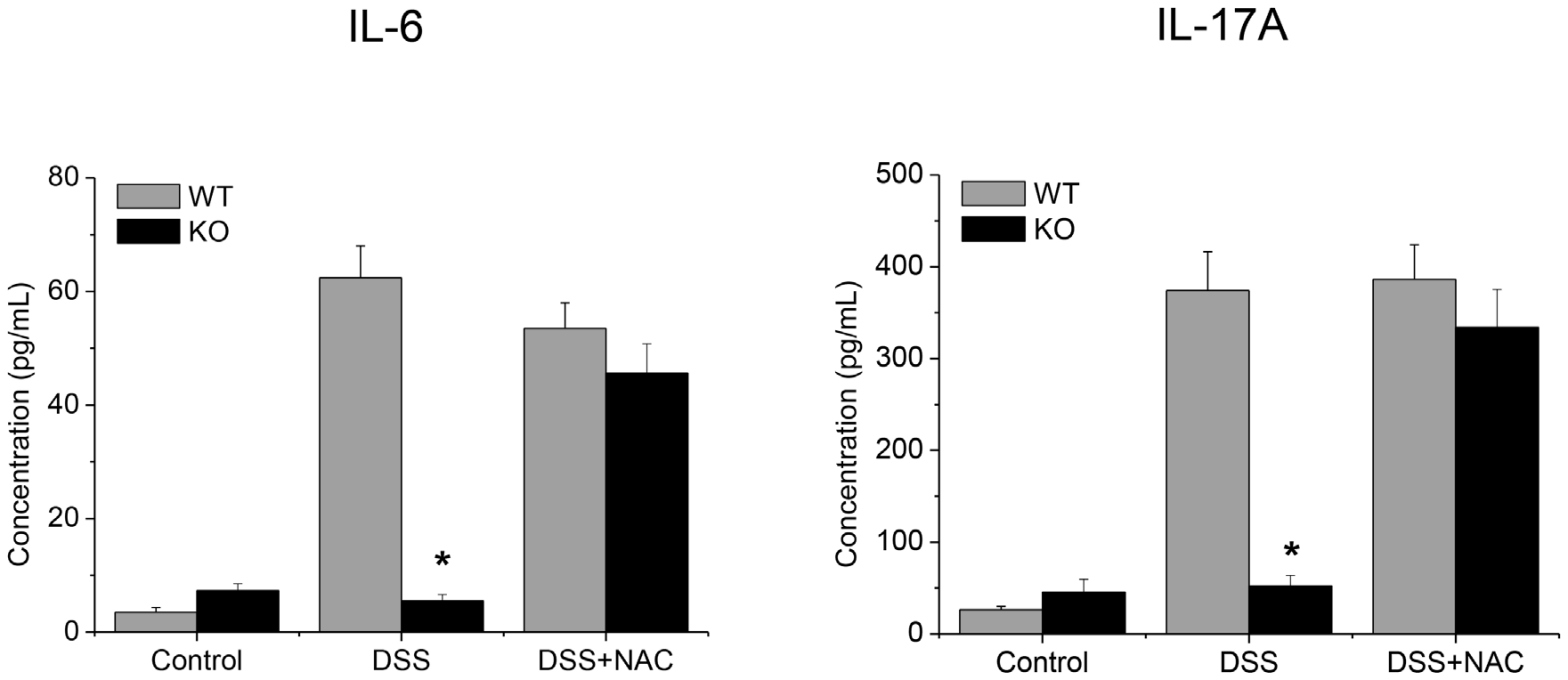
IL-6 and IL-17A production was impaired in KO mice under inflammatory condition but was rescued by administration with N-acetylcysteine (NAC). Splenocytes prepared from the mice treated as indicated were stimulated with anti-CD3e and anti-CD28 for 5 days. IL-6 and IL-17A secreted in the supernatants were analyzed by cytometric bead array. KO, GPx1^−/−^ × Cat^−/−^. Data are mean ± SE of six separate experiments.

In order to investigate the immune responsive pattern, naïve CD4^+^ cells isolated from the spleens of WT or GPx1^−/−^ × Cat^−/−^ mice were induced to differentiate into Th_17_ cells or FoxP3^+^ Tregs by stimulating in the presence of TGF-β1 and IL-6 or IL-2, respectively. The results showed that the proportion of Th_17_ cells differentiated from GPx1^−/−^ × Cat^−/−^ CD4^+^ cells was slightly but significantly lower than that from WT CD4^+^ cells, suggesting less inflammatory tendency of the GPx1^−/−^ × Cat^−/−^ mice (Fig. 10). Actually, the amounts of IL-6 and IL-17A secreted from the GPx1^−/−^ × Cat^−/−^ splenocytes were much less than those from WT splenocytes in DSS-induced colitis (Fig. 11). On the other hand, iTreg differentiation was not significantly different between WT and GPx1^−/−^ × Cat^−/−^ CD4^+^ cells (Fig. S5). Meanwhile, administration of NAC into the GPx1^−/−^ × Cat^−/−^ mice induced secretion of IL-6 and IL-17A, suggesting that NAC affected the GPx1^−/−^ × Cat^−/−^ mice toward pro-inflammatory tendency.

### IDO Expression was Induced in DSS-induced Colitis in WT Mice, while IDO was Expressed from the Beginning in GPx1^−/−^ × Cat^−/−^ Mice

**Figure 12:**
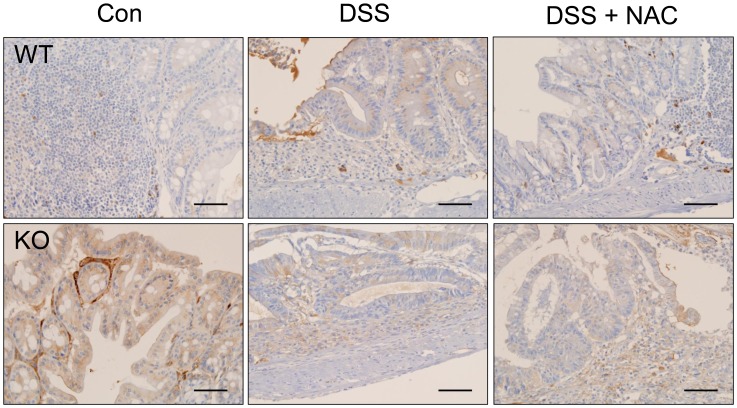
IDO was expressed at steady state from the beginning in KO mice, but was induced after treatment with DSS in WT mice. Immunohistochemical staining of the lesions of DSS-induced colitis for IDO. KO, GPx1^−/−^ × Cat^−/−^. Scale bar is 50 µm.

IDO is an immunoregulatory enzyme that can be induced by ROS [21], [22]. At steady state, IDO expression was rarely observed only in lymphoid follicles in the colons of WT mice (Fig. 12). In the colons of WT mice treated with DSS, IDO expression was observed in the infiltrating cells, and weak expression of IDO was observed in the epithelial cells. By contrast in GPx1^−/−^ × Cat^−/−^ mice, IDO expression was observed in the epithelial cells, and in particular strongly in endothelial cells, at steady state. Meanwhile, in the colons of GPx1^−/−^ × Cat^−/−^ mice treated with DSS, IDO expression was not enhanced, compared with those not treated, although IDO expression was also observed in the infiltrating cells. Addition of NAC also did not enhance IDO expression, although inflammatory reaction was aggravated.

### The Frequency of FoxP3^+^ Tregs was Increased in the Lesions of DSS-induced Colitis in GPx1^−/−^ × Cat^−/−^ Mice

**Figure 13:**
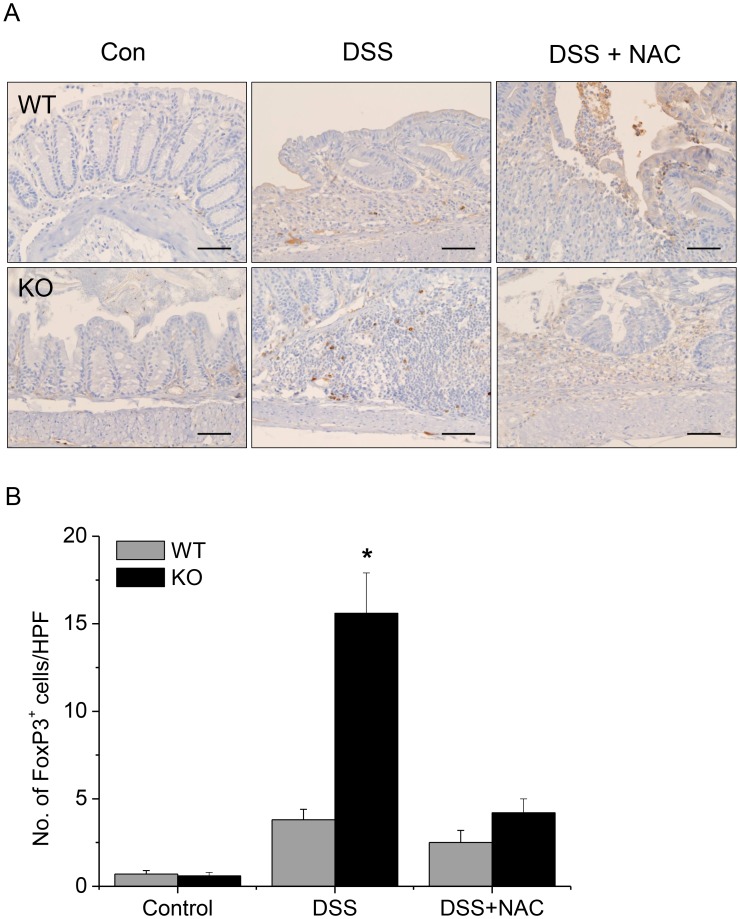
The frequency of FoxP3^+^ Tregs was increased in the lesions of DSS-induced colitis in KO mice. Immunohistochemical staining of the lesions of DSS-induced colitis for FoxP3 (A). For the counting of FoxP3^+^ cells, 5 high-power fields selected at random in each slide were examined by three different pathologists (B). KO, GPx1^−/−^ × Cat^−/−^. Scale bar is 50 µm. Data are mean ± SE of six separate experiments.

FoxP3^+^ cells were hardly observed in the colons of WT mice at steady state (Fig. 13). In the lesions of DSS-induced colitis in WT mice, FoxP3^+^ cells were observed among the infiltrating cells but at a very low frequency. By contrast, in the lesions of DSS-induced colitis in GPx1^−/−^ × Cat^−/−^ mice, FoxP3^+^ cells were observed quite frequently among the infiltrating cells. Meanwhile, the frequency of FoxP3^+^ cells were reduced in the lesions of GPx1^−/−^ × Cat^−/−^ mice treated with both NAC and DSS to the level comparable to WT mice treated with DSS.

## Discussion

In the present study, we demonstrated for the first time that Tregs were hyperfunctional in elevated level of ROS by using GPx1^−/−^ × Cat^−/−^ Tregs. As it has been already reported that Tregs were hypofunctional in lowered levels of ROS [16], [17], it could be argued that Treg function is closely linked to ROS level. Actually in the present study, IP injection of NAC into GPx1^−/−^ × Cat^−/−^ mice reduced the suppressive function of Tregs to the level comparable to WT Tregs (Fig. 8). Administration of NAC also has made GPx1^−/−^ × Cat^−/−^ mice, which are naturally resistant, susceptible to DSS-induced colitis, suggesting the critical role of ROS in the prevention of DSS-induced colitis (Figs. 2–5). The importance of Tregs in the maintenance of intestinal immune balance has been already shown in many other studies [23]. Consequently, ROS level might be critical in the maintenance of intestinal immune homeostasis, providing an insight for the immunomodulation by ROS.

This argument is completely opposing the traditional concept that ROS is involved in the induction and progression of IBD [3]. According to the traditional concept, anti-oxidant intervention should be effective in the treatment of IBD, and large array of antioxidant compounds have been shown to be protective against IBD in experimental animals. Nonetheless, objective clinical data supporting anti-oxidant intervention of human IBD are still scarce [24]. At the moment, it is necessary to establish a new conceptual framework that can compromise the recent observations and the traditional concept. We can draw a clue from the comparison of GPx-1^−/−^ mice and GPx-1^−/−^ × GPx-2^−/−^ mice. As previously mentioned, immune response is attenuated in GPx-1^−/−^ mice [10], [11]. The life span of GPx-1^−/−^ mice is not shortened although cellular DNA damages are accumulated, suggesting that ROS level is elevated, at which the mice are still tolerable [25], [26]. In contrast, inflammatory diseases such as colitis develop spontaneously in GPx-1^−/−^ × GPx-2^−/−^ mice, suggesting that inflammation is augmented [27]. GPx-1^−/−^ × GPx-2^−/−^ mice usually do not grow well and lifespan is shortened, suggesting that ROS level is elevated to a higher level that the mice could not tolerate. Spontaneous development of inflammatory diseases and shortened lifespan observed in GPx-1^−/−^ × GPx-2^−/−^ mice are compatible with the traditional concept on ROS. Therefore, we think that the levels of ROS in many evident previous observations that contribute to establish the traditional concept on ROS, such as vascular reperfusion injury, may be as high as that of GPx-1^−/−^ × GPx-2^−/−^ mice [28], [29]. Thus, we can imagine an arbitrary threshold level of ROS, although we cannot quantitatively specify at the moment, between the higher levels that induce direct tissue damage, such as GPx-1^−/−^ × GPx-2^−/−^ mice, and the moderately high levels at which mice can tolerate, such as GPx1^−/−^mice.

Taken together, we hypothesize, if ROS can induce direct tissue damages at high levels, defensive or compensatory mechanisms counteracting the destructive effects of ROS should be developed in the body. Actually, mice with moderately elevated levels of ROS, such as GPx1^−/−^, PrxII^−/−^ and GPx1^−/−^ × Cat^−/−^ mice, were anti-inflammatory [10], [11], [12], whereas those with lowered ROS level, such as Ncf1^−/−^ and Nox2^−/−^ mice, were pro-inflammatory [5], [6], [7]. From this point of view, it is quite reasonable that the suppressive function of Tregs is closely associated with ROS level. At molecular level, the expression of an immunoregulatory enzyme, IDO, is also associated with ROS level [21], [22]. IDO catabolizes the essential amino acid tryptophan into the stable metabolite, kynurenine [30]. Consequently, IDO depletes tryptophan from the environment, thus starving effector cells. It was also found that tryptophan depletion resulted in inhibition of Th_17_ cell differentiation and expansion of Foxp3^+^ Tregs [22], [31], [32].

IDO is primarily expressed in antigen-presenting cells (APCs), such as dendritic cells and macrophages, but IDO pathway is induced in many tissues during inflammation because IDO gene expression is induced by interferon [22], [33]. In the present study, IDO expression was rarely observed at steady state, but was induced in the epithelial cells as well as in the infiltrating cells in the lesions of DSS-induced colitis (Fig. 12). Thus, IDO expression might be induced as a consequence of the inflammatory reaction, contributing to the feedback regulation. On the other hand, in GPx1^−/−^ × Cat^−/−^ mice, IDO expression was observed in many types of the cells, including endothelial, epithelial and APC-like cells, from the beginning. Elevated levels of ROS not only contribute to the induction but also enhance the enzyme activity of IDO, as superoxide radical acts as a cofactor of IDO [30]. Therefore, high expression and strong activity of IDO from the beginning in GPx1^−/−^ × Cat^−/−^ mice might contribute to the preparation of immunosuppressive environment preventing inflammatory tissue damage during treatment with DSS. Actually in the present study, the frequency of FoxP3^+^ cells was significantly increased in parallel with significantly attenuated inflammatory reaction in the lesions of DSS-induced colitis in mice with elevated level of ROS due to defects in GPx1 and Cat (Fig. 13). By contrast, IP injection of NAC significantly reduced the frequency of FoxP3^+^ cells and aggravated inflammatory reaction in the lesions of DSS-induced colitis. IP injection of NAC also aggravated DSS-induced colitis in WT mice, inducing earlier weight loss and more severe inflammatory changes. This result is quite different from the report by Amrouche-Mekkioui & Djerdjouri (2012) that NAC improved DSS-induced colitis [24]. Meanwhile, the experimental schedules were different as our model was acute colitits while theirs was chronic colitis. However, further investigation is needed to understand the exact reasons of such opposing results.

For the functional assay of Tregs, we used CD4^+^CD25^+^ fraction isolated from the spleens of GPx1^−/−^ × Cat^−/−^ and WT mice. Although Treg was originally identified in CD4^+^CD25^+^ fraction, the fraction is not specific to Tregs [34]. At present, FoxP3 is the most specific to Tregs, but it is not also absolutely specific [35], [36]. Anyway, FoxP3^+^ fraction would be more appropriate than CD4^+^CD25^+^ fraction for the functional assay of Tregs, but it is still technically impossible to isolate FoxP3^+^ cells as live state for functional assay. Because FoxP3 is an intranuclear transcription factor, it is unavoidable to destroy cytoplasmic membrane to stain FoxP3. Of course, it is possible to isolate live FoxP3^+^ cells from special genetically modified mice, such as eGFP-FoxP3 knock-in mice [37]. However, at the moment, they are still unavailable on GPx1^−/−^ × Cat^−/−^ background, instead we used CD4^+^CD25^+^ fraction for the functional assay of Tregs. Efimova et al (2011), who demonstrated Tregs were hypofunctional in lowered levels of ROS, have also used CD4^+^CD25^+^ fraction for the functional assay of Tregs, taking into account the small FoxP3^−^ fraction in the CD4^+^CD25^+^ fraction [16]. We also followed the same method in the present study.

Concerning on the suppressive assay of Tregs, we previously demonstrated that the suppressive patterns of Tregs might be different according to the strength of stimulating signals [19], [38]. Thus, it is necessary to stimulate the cells in a wide range of signal strength for the accurate functional assessment of Tregs. In the present study, we used a variety of concentrations of anti-CD3e antibody for different range of signal strength. Based on our experience of several years on Treg functional assay, we employed a precise quantitative parameter for the proliferative responsiveness, precursor frequency, which represents the fraction of the cells at the initial time point that have gone into cell cycle [20], [39], [40].

In the present study, we demonstrated an experimental colitis was attenuated in elevated level of ROS. Enhancement of Treg function and IDO expression, investigated in the present study, might be involved in the underlying mechanism. However, we cannot exclude the possibility of involvement of other molecules and cells that have not investigated in the present study. Taken together, the results of the present study suggest the potential therapeutic strategy for IBD through immunomodulation by ROS.

## Supporting Information

Figure S1 **Purity of isolated CD4^+^CD25^+^ fraction.** The isolated CD4^+^CD25^+^ fraction is not pure Treg population, in terms of FoxP3 expression. CD4^+^FoxP3^+^ cells ranged from 86.6 ∼ 91.4% (88.2±3.4%, n = 12) in the CD4^+^CD25^+^ fraction.
(TIF)

Figure S2 **KO Tregs were hyperfunctional.** CFSE-labeled Teffs were stimulated with various concentrations of soluble anti-CD3e as indicated in the presence of DCs and Tregs from WT or KO mice. On the 3^rd^ day, the cells were harvested and stained for surface CD4. Live CD4^+^CFSE^+^ cells were gated for the analysis of the proliferative responsiveness of Teffs. The prolifeative response of Teffs in the presence of KO Tregs (row 2, 4, 6, 8) was less active than in the presence of WT Tregs (row 1, 3, 5, 7), suggesting KO Tregs were hyperfunctional in the suppression of Teffs than WT Tregs. KO, GPx1^−/−^ × Cat^−/−^. Numbers indicate precursor frequency (%) of Teffs representing proliferative activity. A representative series of FACS plots of six separate experiments showing the same pattern.
(TIF)

Figure S3 **KO Teffs were hyperproliferative than WT Teffs.** CFSE-labeled Teffs were stimulated with various concentrations of soluble anti-CD3e as indicated in the presence of DCs from WT or KO mice. On the 3^rd^ day, the cells were harvested and stained for surface CD4. Live CD4^+^CFSE^+^ cells were gated for the analysis of the proliferative responsiveness of Teffs. The prolifeative response of Teffs was slightly more vigorous than WT Teffs in the presence of WT DCs, but not in the presence of KO DCs. KO, GPx1^−/−^ × Cat^−/−^. Numbers indicate precursor frequency (%) of Teffs representing proliferative activity. A representative series of FACS plots of six separate experiments showing the same pattern.
(TIF)

Figure S4 **KO Teffs were hyperproliferative than WT Teffs.** The prolifeative response of Teffs was slightly more vigorous than WT Teffs in the presence of WT DCs, but not in the presence of KO DCs. KO, GPx1^−/−^ × Cat^−/−^. Data are mean ± SE of six separate experiments.
(TIF)

Figure S5 **Inducible (i) Treg differentiation was comparable in KO mice.** Naïve CD4^+^ cells isolated from the spleens of WT or KO mice were induced to differentiate into iTregs by stimulating in the presence of TGF-β1 and IL-2. iTreg differentiation from KO CD4^+^ cells seemed slightly enhanced but not significant. KO, GPx1^−/−^ × Cat^−/−^. Data are mean ± SE of six separate experiments.
(TIF)
