# Supplementary figures and images for: Attenuation of Experimental Colitis in Glutathione Peroxidase 1 and Catalase Double Knockout Mice through Enhancing Regulatory T Cell Function
Source: PLoS One. 2014 Apr 17;9(4):e95332. doi: 10.1371/journal.pone.0095332 (PMC3990669; doi:10.1371/journal.pone.0095332)

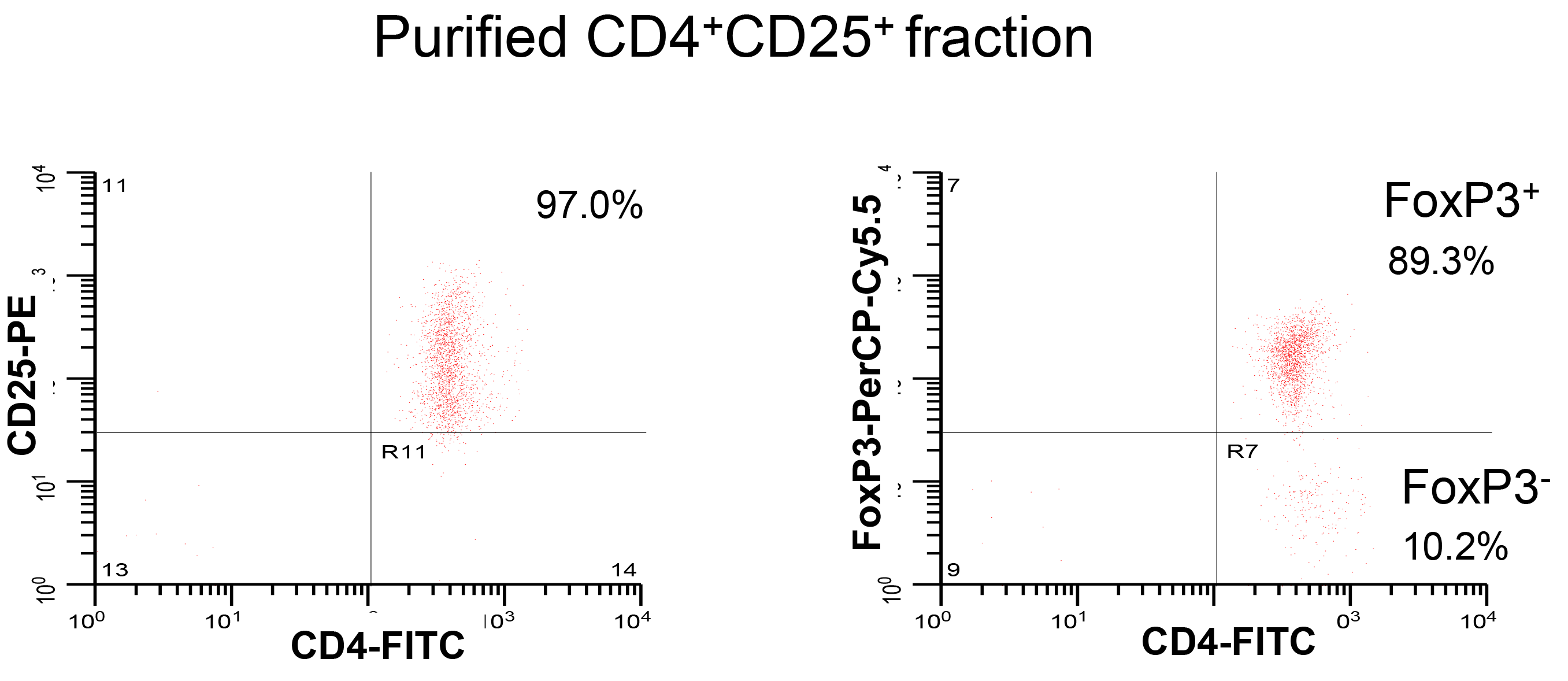

Supplement: Figure S1 — Purity of isolated CD4+CD25+ fraction. The isolated CD4+CD25+ fraction is not pure Treg population, in terms of FoxP3 expression. CD4+FoxP3+ cells ranged from 86.6 ∼ 91.4% (88.2±3.4%, n = 12) in the CD4+CD25+ fraction. (TIF) [file pone.0095332.s001.tif]

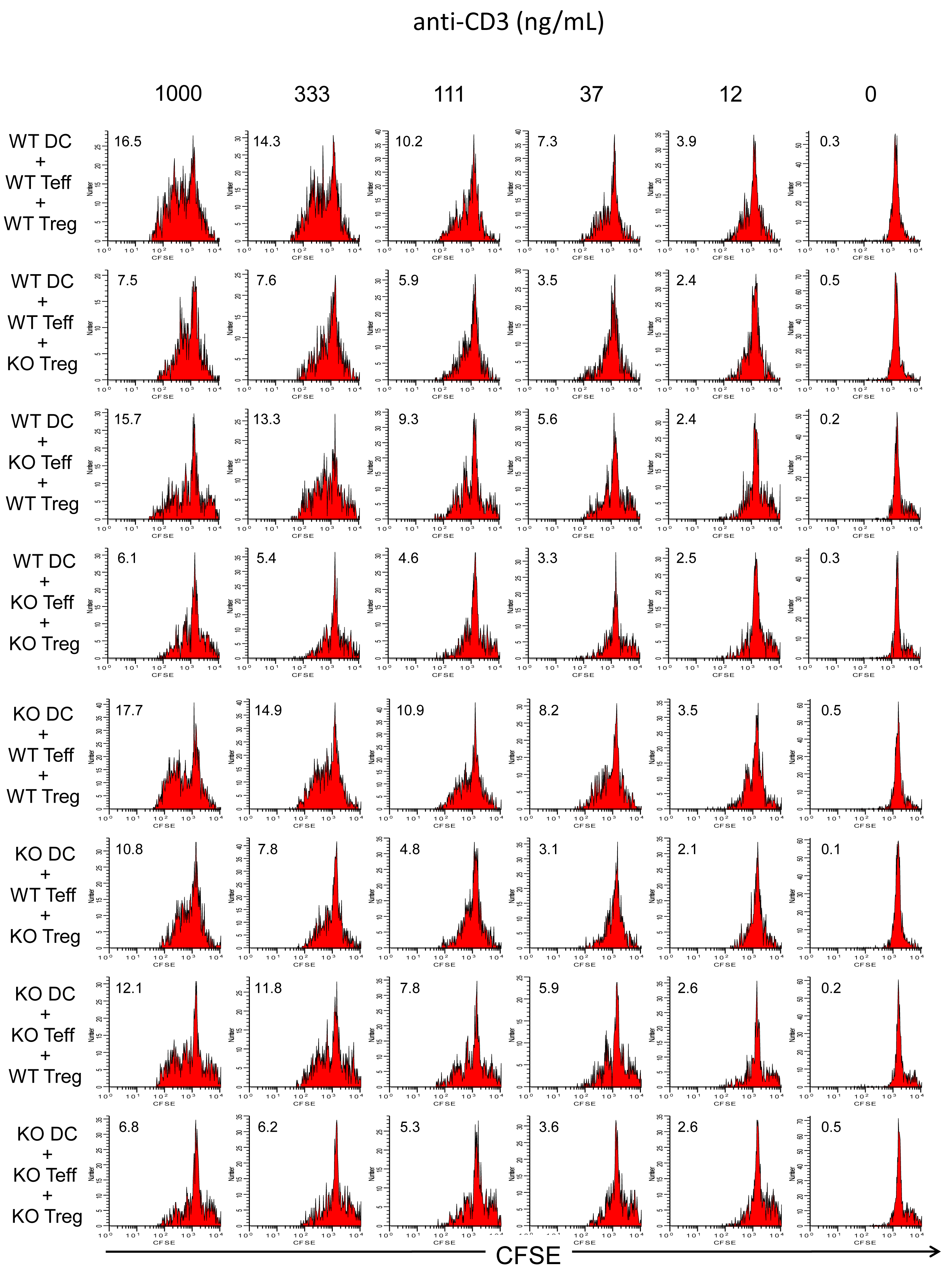

Supplement: Figure S2 — KO Tregs were hyperfunctional. CFSE-labeled Teffs were stimulated with various concentrations of soluble anti-CD3e as indicated in the presence of DCs and Tregs from WT or KO mice. On the 3rd day, the cells were harvested and stained for surface CD4. Live CD4+CFSE+ cells were gated for the analysis of the proliferative responsiveness of Teffs. The prolifeative response of Teffs in the presence of KO Tregs (row 2, 4, 6, 8) was less active than in the presence of WT Tregs (row 1, 3, 5, 7), suggesting KO Tregs were hyperfunctional in the suppression of Teffs than WT Tregs. KO, GPx1−/− × Cat−/−. Numbers indicate precursor frequency (%) of Teffs representing proliferative activity. A representative series of FACS plots of six separate experiments showing the same pattern. (TIF) [file pone.0095332.s002.tif]

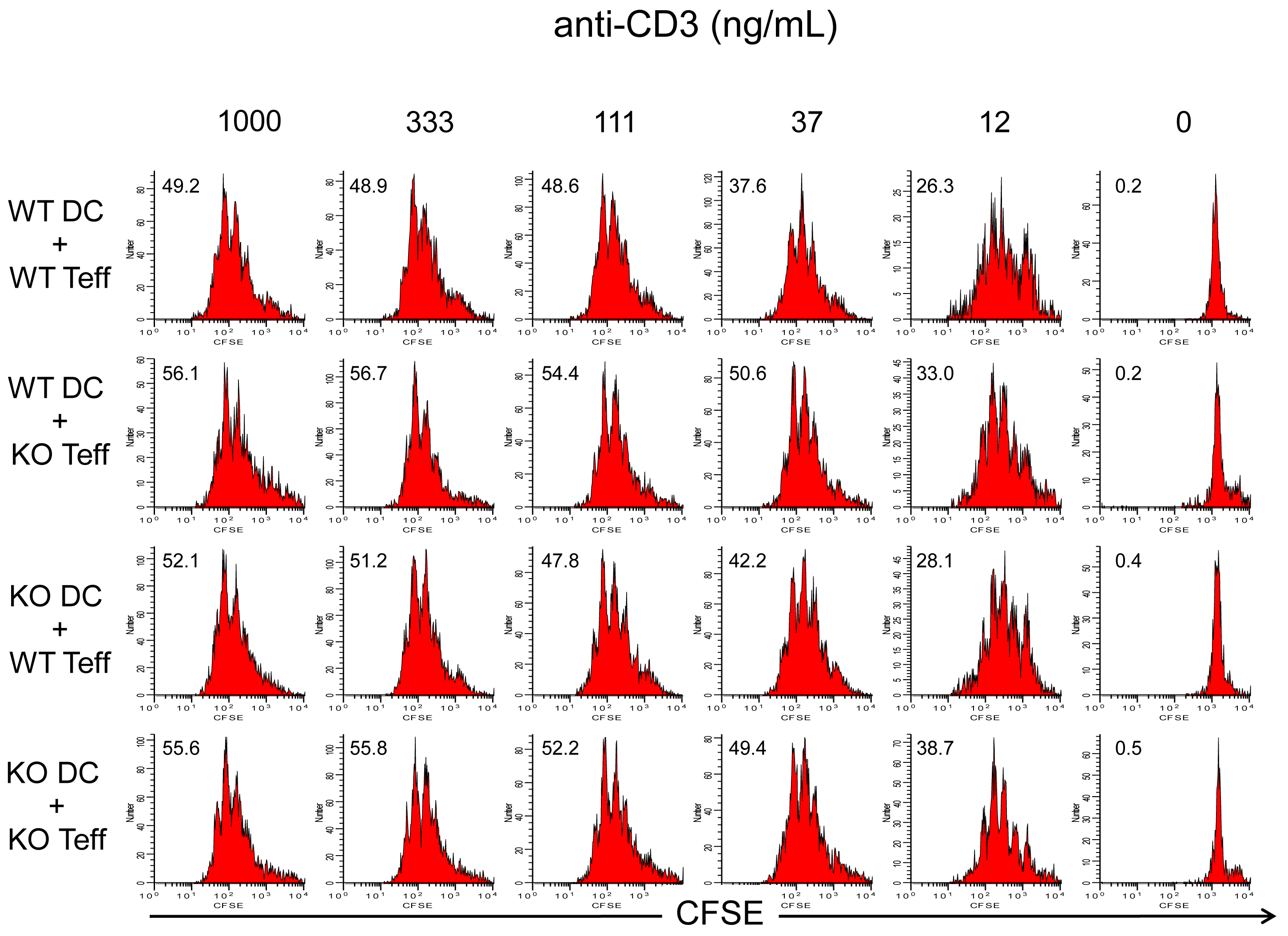

Supplement: Figure S3 — KO Teffs were hyperproliferative than WT Teffs. CFSE-labeled Teffs were stimulated with various concentrations of soluble anti-CD3e as indicated in the presence of DCs from WT or KO mice. On the 3rd day, the cells were harvested and stained for surface CD4. Live CD4+CFSE+ cells were gated for the analysis of the proliferative responsiveness of Teffs. The prolifeative response of Teffs was slightly more vigorous than WT Teffs in the presence of WT DCs, but not in the presence of KO DCs. KO, GPx1−/− × Cat−/−. Numbers indicate precursor frequency (%) of Teffs representing proliferative activity. A representative series of FACS plots of six separate experiments showing the same pattern. (TIF) [file pone.0095332.s003.tif]

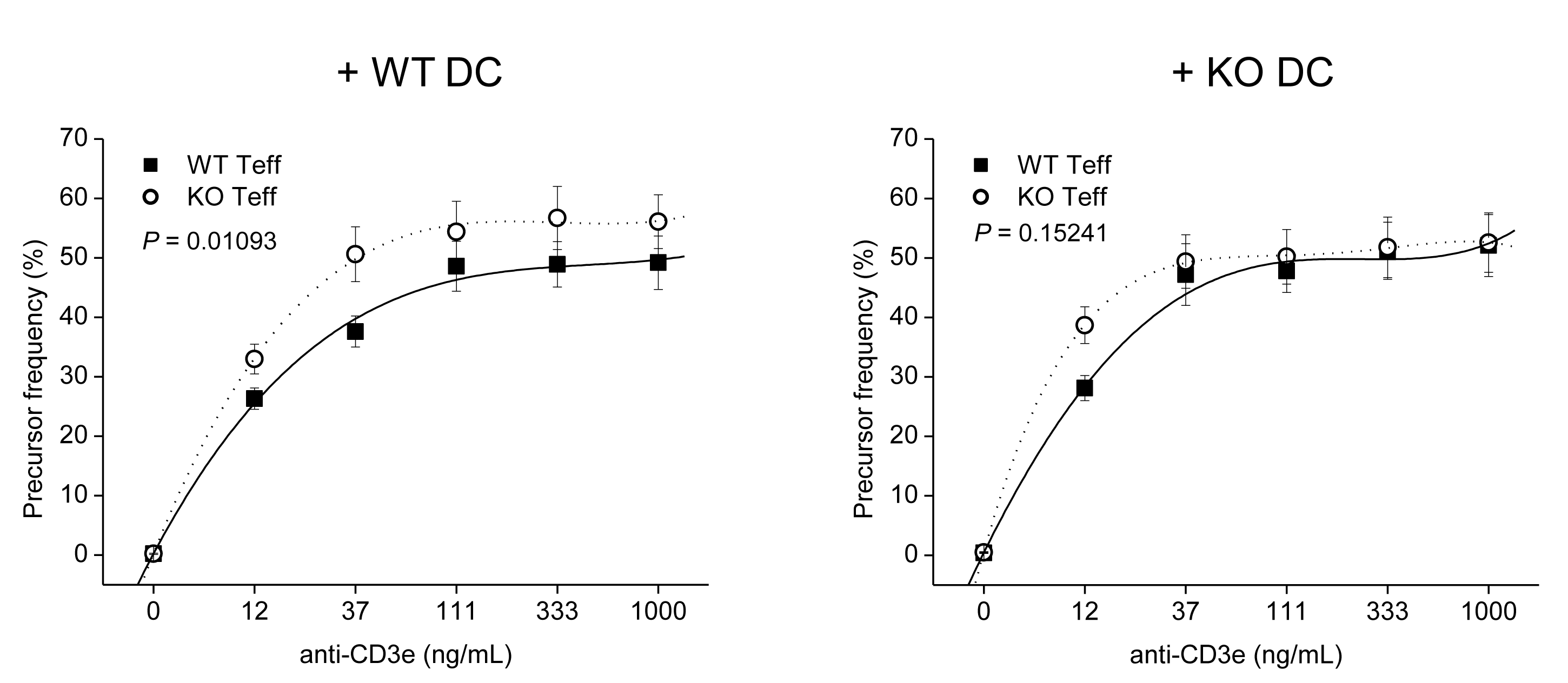

Supplement: Figure S4 — KO Teffs were hyperproliferative than WT Teffs. The prolifeative response of Teffs was slightly more vigorous than WT Teffs in the presence of WT DCs, but not in the presence of KO DCs. KO, GPx1−/− × Cat−/−. Data are mean ± SE of six separate experiments. (TIF) [file pone.0095332.s004.tif]

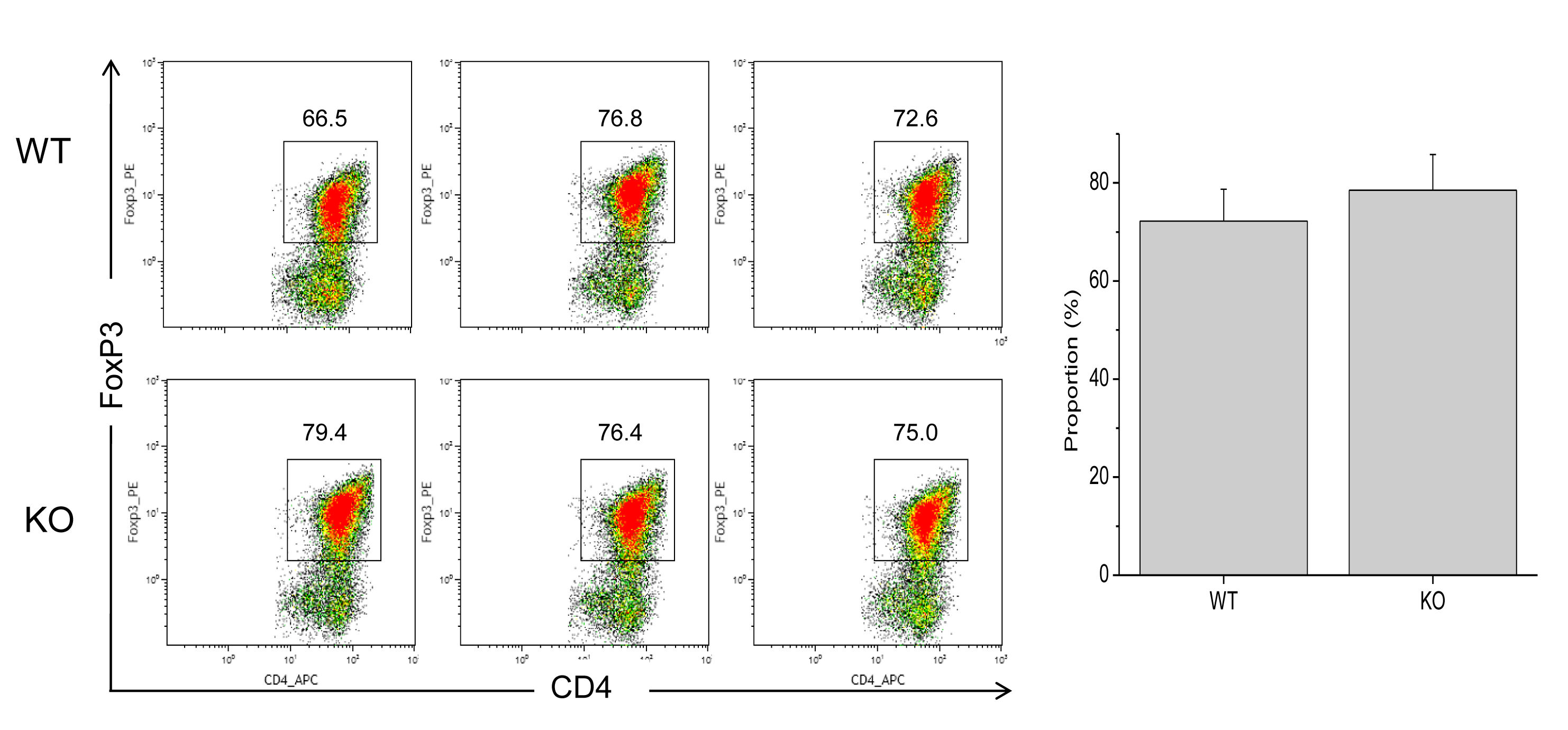

Supplement: Figure S5 — Inducible (i) Treg differentiation was comparable in KO mice. Naïve CD4+ cells isolated from the spleens of WT or KO mice were induced to differentiate into iTregs by stimulating in the presence of TGF-β1 and IL-2. iTreg differentiation from KO CD4+ cells seemed slightly enhanced but not significant. KO, GPx1−/− × Cat−/−. Data are mean ± SE of six separate experiments. (TIF) [file pone.0095332.s005.tif]
